# Valorization of By-Products from Biofuel Biorefineries: Extraction and Purification of Bioactive Molecules from Post-Fermentation Corn Oil

**DOI:** 10.3390/foods11020153

**Published:** 2022-01-07

**Authors:** Francesco Cairone, Stefania Cesa, Alessia Ciogli, Giancarlo Fabrizi, Antonella Goggiamani, Antonia Iazzetti, Gabriella Di Lena, Jose Sanchez del Pulgar, Massimo Lucarini, Luca Cantò, Gokhan Zengin, Petra Ondrejíčková

**Affiliations:** 1 Department of Drug Chemistry and Technology, Sapienza, University of Roma, P.le A. Moro 5, 00185 Rome, Italy; francesco.cairone@uniroma1.it (F.C.); stefania.cesa@uniroma1.it (S.C.); alessia.ciogli@uniroma1.it (A.C.); giancarlo.fabrizi@uniroma1.it (G.F.); antonella.goggiamani@uniroma1.it (A.G.); 2 Dipartimento di Scienze Biotecnologiche di Base, Cliniche Intensivologiche e Perioperatorie, Università Cattolica del Sacro Cuore, L.go Francesco Vito 1, 00168 Rome, Italy; 3 CREA Research Centre for Food and Nutrition, Via Ardeatina 546, 00178 Rome, Italy; jsapuri@hotmail.com (J.S.d.P.); massimo.lucarini@crea.gov.it (M.L.); 4 Department of Pharmacy, University “G. d’Annunzio” of Chieti-Pescara, 66100 Chieti, Italy; luca_canto@yahoo.it; 5 Department of Biology, Science Faculty, Selcuk University, 42130 Konya, Turkey; gokhanzengin@selcuk.edu.tr; 6 ENVIRAL a.s., Trnavská Cesta, 920 41 Leopoldov, Slovakia; Ondrejickova@enviengroup.eu

**Keywords:** post-fermentation corn oil, phenolics, carotenoids, bioactive molecules, valorization of industrial side streams, antioxidant activity

## Abstract

The aim of this work was to develop innovative and sustainable extraction, concentration, and purification technologies aimed to recover target substances from corn oil, obtained as side stream product of biomass refineries. Residues of bioactive compounds such as carotenoids, phytosterols, tocopherols, and polyphenols could be extracted from this matrix and applied as ingredients for food and feeds, nutraceuticals, pharmaceuticals, and cosmetic products. These molecules are well known for their antioxidant and antiradical capacity, besides other specific biological activities, generically involved in the prevention of chronic and degenerative diseases. The project involved the development of methods for the selective extraction of these minor components, using as suitable extraction technique solid phase extraction. All the extracted and purified fractions were evaluated by NMR spectroscopic analyses and UV–Vis spectrophotometric techniques and characterized by quali-quantitative HPLC analyses. TPC (total phenolic content) and TFC (total flavonoid content) were also determined. DPPH and ABTS radical were used to evaluate radical quenching abilities. Acetylcholinesterase (AChE), amylase, glucosidase, and tyrosinase were selected as enzymes in the enzyme inhibitory assays. The obtained results showed the presence of a complex group of interesting molecules with strong potential in market applications according to circular economy principles.

## 1. Introduction

With the perspective of an increasing world population, expected to exceed 9 billion people by 2050, the current production and consumption patterns, relying on extensive exploitation of natural resources, are no longer sustainable. To decouple the economic growth from environmental impact and depletion of resources, the adoption of a new economic model based on circularity becomes urgent [1].

The recovery and valorization of agri-food and agro-industrial waste and by-products via the extraction of bioactive compounds, for further production of functional products for high-value markets, is a promising strategy able to give impulse to the economy while reducing the loss of resources and energy as also the environmental burden represented by waste disposal [2,3,4].

A particular kind of by-product is represented by those generated by biofuel biorefineries. These side streams are produced in large volumes and are rich in nutrients and valuable biomolecules that, if properly recovered and valorized, may maximize the efficiency and competitiveness of biofuel production processes.

Corn is an important crop produced in large quantities all over the world. Commercial corn oil is mainly obtained by the germ or the kernels of *Zea mays* L., the main world producers being the United States of America, Mexico, Russia, Belgium, France, Germany, Spain, Italy, and the United Kingdom [5].

Besides being an important staple food all over the world, corn is also the most common feedstock for bioethanol production in Europe, where over 49% of the bioethanol produced (corresponding to 2.76 billion L/year) comes from the biotechnological processing of corn [6].

One co-product of dry-grind corn bioethanol biorefineries is post-fermentation corn oil, separated by centrifugation from a corn syrup obtained after enzymatic hydrolysis, fermentation, and distillation processes. Previous studies have highlighted the chemical composition and valorization potentials of dry-grind corn bioethanol biorefinery side streams [7,8]. In particular, post-fermentation corn oil is rich in plant sterols and stanols and also maintains the full set of tocopherols, tocotrienols, and carotenoids derived from the corn kernel as well as from yeast.

As this oil is currently utilized as biodiesel feedstock, these bioactives, with their antioxidant, anti-inflammatory, hypocholesterolemic, anti-aging, and several other health-promoting potentials, remain essentially unutilized. With a circular economy approach, the bioactive molecules present in corn bioethanol oil (plant sterols, stanols, tocopherols, tocotrienols, carotenoids, and phenolic acids) may be recovered through appropriate technologies with the final aim to re-introduce them in productive processes as ingredients of bio-based functional products for high-value (food, nutraceutic, cosmetic) markets. Finally, the same triglycerides, which represent almost all corn oil, deserve particular attention. In fact, these could be newly employed as raw material both in the production of biodiesel and, once purified by eventual contaminants, for food, feed, or cosmetic use, minimizing environmental impact and waste. In fact, the growing consumers’ concern for safe and natural products is a potent market driver in the food and health-care sectors for the development of innovative and healthy products. In particular, there is a growing demand for protein and bioactive molecules as ingredients for functional products, particularly those targeted to the special requirements of elderly people, sport nutrition, and infants. Solid phase extraction (SPE) is commonly used for pre-concentration and separation of bioactive compounds from natural matrices, allowing a selective binding of analytes with minimal solvent consumption and high flexibility.

The aim of this work was to investigate the possibility to reap the full potential of post-fermentation corn oil, enhancing its white biotechnology applications. In accordance with the European policy objectives of circular economy and consequent reduction in environmental impact, we pointed at the development of an innovative and highly sustainable extraction/purification/concentration technology to be applied for the recovery of carotenoid, phytosterols, and polyphenols from biofuel biorefinery corn oil in view of their application in high-value market products (food, nutraceutics, cosmetics, or pharmaceutical formulations).

## 2. Materials and Methods

### 2.1. Materials

Post-fermentation corn oil was procured from ENVIRAL a.s (Leopoldov, Slovakia), an industrial dry-grind corn bioethanol plant. A yellow non-genetically modified corn (*Zea mays*) grown in Central East Europe in 2018 was the original feedstock. The production processes and chemical composition of bioethanol corn oil have been previously reported [3].

The employed solvents (HPLC grade), drying agents, and chemicals were obtained from Carlo Erba reagents S.r.l (Milano, Italy) and from Merck life Science S.r.l (Milano, Italy). Silica gel was obtained from Macherey Nagel (Düren, Germany), Celite and Alumine from Merck life Science s.r.l (Milano, Italy). Pure standards of phenolic acids, tocopherols, ergosterol, stigmasterol, campesterol, β-sitosterol, δ-5-avenasterol, squalene, β-carotene, β-sitosterol, all-trans lutein, all-trans zeaxanthin, and all-trans β-cryptoxanthin were purchased from Merck life Science S.r.l (Milano, Italy) and Sigma-Aldrich Inc (St. Louis, MO, USA). Sitostanol was from AVANTI Polar Lipids Inc. (Alabaster, AL, USA). Tocotrienols were purchased from Cayman Chemical Company (Ann Arbor, MI, USA). Potassium hydroxide and Butylated hydroxytoluene (BHT) were from Carlo Erba. Tert-butyl-hydroquinone (TBHQ) was from Fluka Chemie AG (Buchs, Switzerland). Deionised water was provided by an Arium^®^ pro UV Water Purification System (Sartorius Stedim Biotech GmbH, Goettingen, Germany).

### 2.2. Sample Treatment

**Figure 1:**
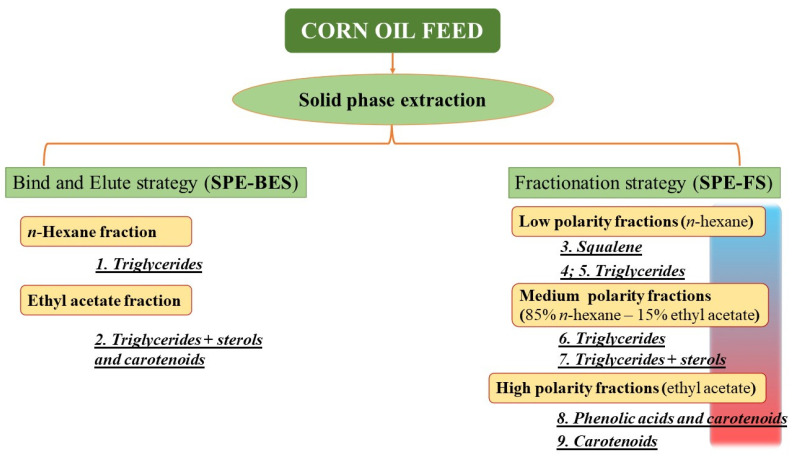
Work-up and the corresponding fractions.

Upon arrival at the laboratory, post-fermentation corn oil was stored refrigerated at 4 °C and preserved from light. Corn oil feed was processed according to Figure 1. Before analyses, the oil was placed to room temperature and mildly shaken in order to re-suspend any eventual sediment.

### 2.3. Solid Phase Extraction (SPE)

SPE extractions have been performed according to two main strategies: a bind and elute strategy (SPE-BES) or a fractionation strategy (SPE-FS). Both processes were performed utilizing the same experimental procedure, which consisted of an extractive axial stainless column (50.0 mm × 40.0 cm) equipped with stainless steel sintered filter disk, filled with 150 g of SiO_2_ suspended into 500 mL of *n*-Hexane.

Corn oil (40.0 g) was dissolved into 40 mL of *n*-Hexane, loaded onto the column, and eluted by applying a pressure of 2.5 bar generated by a solvent delivery system (Jasco PU2087, preparative HPLC pump, Cremella, Italy).

With the SPE-BES, two fractions consisting of 2500 mL of *n*-hexane and 2500 mL of ethyl acetate (AcOEt), respectively, were collected (1 and 2), dried under pressure at 35 °C, weighed, and stored at −20 °C until the analyses were performed.

With the SPE-FS, the extraction process was performed employing sequentially three different eluents (1500 mL of *n*-hexane, 1500 mL of a mixture *n*-hexane/AcOEt 85/15 *v/v*, and 1500 mL of AcOEt) at a flow rate of 25 mL/min. Three different low-polarity (3, 4, 5), two medium-polarity (6, 7), and two high-polarity fractions (8, 9) were collected, dried, weighed, and stored at −20 °C until the analyses were performed.

### 2.4. Purification Step

#### 2.4.1. Phytosterols Purification

Fractions 7 and 8 (1.00 g) were suspended in *n*-hexane (3 mL) at 4 °C overnight. The precipitate obtained (7a and 8a, respectively) was collected after centrifugation (10 min at 112 G), dried, weighed, and stored at −20 °C until the analyses were performed.

#### 2.4.2. Selective Extraction of Carotenoids on RP18 Silica Gel

**Figure 2:**
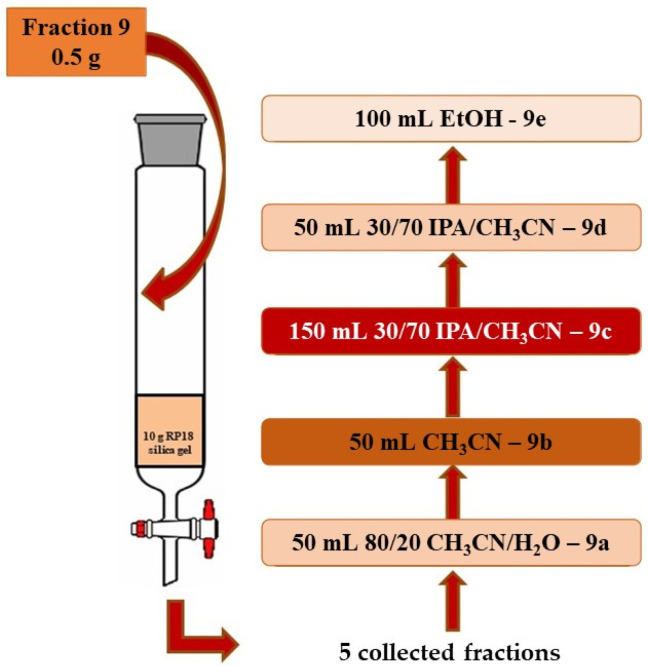
Selective extraction of carotenoids by RP-C18-SPE of fraction 9.

About 0.50 g of fraction 9 were chromatographed on a 10 g RP18 silica gel (particle size: 20–45 µm), eluting with different mobile phases in the following order: 50 mL of acetonitrile/H2O, 80/20 *v*/*v* (fraction 9a); 50 mL of acetonitrile (fraction 9b); 150 mL of isopropanol (IPA)/acetonitrile, 30/70 *v*/*v* (fraction 9c); 50 mL of isopropanol (IPA)/acetonitrile, 30/70 *v*/*v* (fraction 9d); 50 mL of ethanol (fraction 9e), see Figure 2 fractions 9a–e were collected, dried, weighed, and stored at −20 °C until the analyses were performed.

### 2.5. High-Performance Liquid Chromatography (HPLC) Analysis

HLPC-DAD analyses were carried out using a 1100 Series Agilent HPLC System (Agilent Technologies, Santa Clara, CA, USA) equipped with a quaternary pump, a solvent degasser, a column thermostat, and photodiode-array (DAD) detector.

Tocopherols, tocotrienols, plant sterols, and squalene were determined simultaneously on a reversed-phase Ultrasphere C-18 column (25 cm × 4.6 mm inner diameter, 5 m, Beckman, Palo Alto, CA, USA) coupled with a C18 guard column (15 cm × 4.6 mm, 5 µm). The mobile phase consisted of acetonitrile/methanol (50:50, *v/v*) in isocratic conditions at a flow rate of 1.5 mL min^−1^ at 25 °C. Runs were monitored at 215 nm and 282 nm.

Carotenoids were separated using a reversed-phase C-30 column (25 cm × 4.6 mm inner diameter, 5 µm) coupled with a C30 guard cartridge (10 mm, 4 mm, particle size 5 μm), both from YMC Co., Ltd. (Basel, Switzerland). The mobile phase consisted of methanol (eluent A), methyl tert-butyl ether (eluent B), and water (eluent C) according to the following gradient: time 0: 81% A—15% B—4% C, time 90 min: 7% A—90% B—3% C. Chromatographic conditions were as described previously [8]. Injection volume was 20 µL. Carotenoids were integrated at 450 nm.

Analytes were identified by comparing retention times and UV–Vis absorption spectra to those of authentic standards. Peak areas were used to calculate analyte concentrations in the samples by reference to standard curves attained by pure substances chromatography, under identical conditions. External linear calibration curves of analytical standards, with a minimum of five concentration levels, were built for each analyte. The DAD response for each analyte was linear within the calibration ranges with correlation coefficients exceeding 0.998. Repeatability was estimated by calculating the coefficient of variation (CV) after replicated runs of a standard solution containing each compound at the level found in samples. CV values for all compounds were below 3%. After HPLC runs, the purity of analytes was checked by matching the UV/Vis spectra of each peak with those of the standards. Data were analyzed with the Agilent ChemStation Software.

### 2.6. High-Performance Liquid Chromatography Coupled to Mass Spectrometer (LC–MS/MS)

The fractions were also analyzed in the LC–MS/MS system in order to improve the identification of the detected compounds. Analyses were performed on an Agilent 1200 quaternary pump coupled to a 6410 series triple quadrupole. The ion source was an APCI operating in positive mode. Chromatographic separation was conducted on an ACME C18-120A, 100 mm × 2.1 mm column, with 3 µm particle size. Mobile phase was the same reported for carotenoid analyses in the previous paragraph, at a flow rate of 300 µL min^−1^. The APCI ionization parameters were as follows: gas temperature 350 °C, vaporizer 375 °C, gas flow 6 L min^−1^, nebulizer 60 psi, capillary voltage 3000 V, and corona current 8 µA. Post-fermentation corn oil polar fractions were analyzed for phenolic acids content by means of LC-MS/MS with an ESI source operated in negative mode. Separation was performed in a core–shell Halo C18 column (100 × 2.1 mm, 90 Å, 2.7 µm) provided by Advanced Materials Technology (Wilmington, DE, USA) using 0.05% acetic acid in water (A) and 0.05% acetic acid in acetonitrile (B) at a mobile phase flow of 0.4 mL min^−1^ and applying the following elution gradient: initial 98% phase A, hold for 2 min, increase phase B to 37% at minute 15, further increase to 80% at minute 18, hold for 2 min, and return to initial conditions at minute 22. The column was then re-equilibrated for 10 min (post-run) before the successive injection. Data acquisition was accomplished in the d-MRM mode with two transitions for each compound. Quantification was carried out by means of external calibration, with a calibration curve of pure standard compounds injected in the same chromatographic conditions, in the range of 25 to 2000 µg/L.

### 2.7. Semipreparative HPLC-Refractive Index Detector

Fraction 8 was dissolved in dichloromethane, filtered, and injected into an HPLC semipreparative apparatus, consisting of a Waters (Milford, MA, USA) Millipore 150 pump, a Gilson (Middleton, WI, USA) 132 refractive index detector, and Jasco (Easton, MD, USA). Borwin software was used for the data acquisition. Chromatography was performed on a Macherey-Nagel, (Bethlehem, PA, USA) 100–5 column. The eluent mixture used was 85/15 (*v/v*) *n*-hexane/ethyl acetate and the flow rate was fixed at 5 mL/min. The analysis was performed at 25 °C.

### 2.8. ^1^H- and ^13^C-NMR Analysis

^1^H and ^13^C NMR (400.13 and 100.03 MHz) analyses were recorded with a Bruker (Billerica, MA, USA) Avance 400 (Milano, Italy) spectrometer, equipped with a Nanobay console and Cryoprobe Prodigy Probe. About 20 mg of the analyzed samples were dissolved in 0.6 mL of CDCL_3_ (I.E% = 99.80%), transferred to an NMR tube, and analyzed. The resulting ^1^H NMR and ^13^C NMR spectra were processed using Bruker TOPSPIN TopSpin 3.5pl2 software.

### 2.9. Total Phenolic Content (TPC) Evaluation

The TPC was defined through a modified Folin–Ciocâlteu method [5] using a 96-well plate (Thermo-Multiskan, Thermo Fisher Scientific, Vantaa, Finland). Each sample (50 μL) was mixed with diluted Folin–Ciocalteu reagent (100 μL, 1:9, *v/v*) and then added sodium carbonate (2%, 75 μL). The mixture was incubated in the dark for 2 h at room temperature. Then, the absorbances were read at 765 nm. Gallic acid was used as a standard, and the results were evaluated as gallic acid equivalent (mg GAE/g sample).

### 2.10. Total Flavonoid Content (TFC) Evaluation

The TFC was detected by the method reported by [6]. A 200 μL sample was mixed with the AlCl_3_ (2% in methanol). The mixture was mixed and incubated for 15 min at room temperature. Then, the absorbances were read at 415 nm. Rutin was used as a standard flavonoid, and the results were evaluated as equivalent of rutin (mg RE/g sample).

### 2.11. DPPH Radical Scavenging Assay

The sample (50 μL) was mixed with methanolic DPPH solution (0.004%), and then the mixture was incubated for 30 min at room temperature. The absorbances were read at 517 nm. Trolox was used as a standard antioxidant, and the results were expressed as equivalent of Trolox (mg TE/g sample) [9].

### 2.12. Trolox Equivalent Antioxidant Capacity (TEAC) Assay

The radical scavenging activity of samples toward the ABTS radical cation was evaluated according to Mocan et al., 2016 [10]. The prepared ABTS radical solution was used after 12 h incubation at room temperature (7 mM ABTS solution was mixed with 2.45 mM potassium persulfate). Firstly, the prepared ABTS solution was diluted with methanol to an absorbance of 0.700 ± 0.02 at 734 nm. The sample (25 μL) was mixed with the prepared ABTS radical (200 μL), and after 30 min at room temperature, the absorbances were recorded at 734 nm. Trolox was used as a standard antioxidant, and the results were expressed as equivalent of Trolox (mg TE/g sample).

### 2.13. α-Amylase Inhibitory Activity

Sample solution was mixed with α-amylase solution (ex-porcine pancreas, EC 3.2.1.1, Sigma) (50 μL) in phosphate buffer (pH 6.9 with 6 mM sodium chloride) in a 96-well microplate and incubated for 10 min at 37 °C. Then, the reaction was initiated with the addition of starch solution (50 μL, 0.05%). The mixture was incubated (10 min, at 37 °C). HCl (25 μL, 1 M) was added to stop reaction, and then the iodine-potassium iodide solution (100 μL) was added. The absorbances were read at 630 nm. Acarbose was used as a positive control, and the results were evaluated as equivalent of acarbose (mmol ACE/g sample) [11].

### 2.14. Tyrosinase Inhibitory Activity

Sample solution (50 µL) was mixed with tyrosinase solution (40 μL) in phosphate buffer (40 mM, pH 6.8) and the mixture was incubated for 15 min at 25 °C. Then, L-DOPA was added to start enzymatic reaction. After 10 min, the absorbances were recorded at 492 nm. Kojic acid was used as positive control, and the results were evaluated as equivalent of kojic acid (mg KAE/g sample) [12].

### 2.15. Cholinesterase Inhibitory Activity

A 100 µL sample solution was mixed with DTNB (5,5-dithio-bis(2-nitrobenzoic) acid, 125 µL). Then, enzyme solution (AChE or BChE) was added, and the mixture was incubated for 15 min at room temperature. After that, the substrate (ATCI or BTCl) was added. After 10 min incubation, the absorbances were read at 405 min. Galantamine was used as a positive control and the results were evaluated as equivalent of galantamine (mg GALAE/g sample) [13].

### 2.16. α-Glucosidase Inhibitory Activity

A Sample solution (50 µL) was mixed with α-glucosidase solution (from *Saccharomyces cerevisiae*, EC 3.2.1.20, Sigma) (50 µL) and substrate (PNPG: 4-Nitrophenyl-α-D-glucopyranoside, Sigma) (50 µL). After 15 min, sodium carbonate (2%, 50 µL) was added to stop the reaction. The absorbances were read at 405 nm. Acarbose was used as a positive control, and the results were evaluated as equivalent of acarbose (mmol ACE/g sample) [13].

### 2.17. Statistical Analysis

Antioxidant and enzyme inhibitory assays were performed in triplicate (mean ± standard deviation). To determine differences in the tested samples, we performed student *t*-test (*p* < 0.05) in Xlstat 2018 software.

## 3. Results and Discussion

### 3.1. Solid Phase Extraction (SPE) and HPLC Analysis

Solid phase extraction (SPE), generally recognized as a cost effective and versatile method, can represent a very selective and effective technique only if the aim is the separation of components quite different in polarity properties. It does not represent, on the contrary, the choice method in other cases [14].

SPE has been performed according to two main strategies. Under the condition of “bind and elute strategy” (SPE-BES), valuable compounds are expected to bind the sorbent material, while unwanted matrix components are washed off. Then, eluting with specific solvents, which disrupt the analyte and adsorbent interaction, only chemicals of interest could be removed from stationary phase. Otherwise, according to the “fractionation strategy” (SPE-FS), different classes of compounds adsorbed on the stationary phase could sequentially eluted by modifying the eluent composition. In SPE-BES, two different solvents were used as eluent: a *n*-hexane fraction (1) corresponding to about 36% of the loaded sample and an ethyl acetate fraction (2), which accounted for the remaining about 63%. The use of *n*-hexane that resulted was not appropriate for the complete elution of triglycerides. Moreover, the composition of the two obtained fractions was quite similar, as shown by the NMR spectra (Figure S1, Supplementary Materials), both fractions retaining high triglycerides amounts, which makes it difficult to apply further purification steps.

The expansion of ^13^C NMR spectrum of fraction 2 (Figure S2, Supplementary Materials) shows the coelution of sterols and carotenoids within the triglycerides fraction. To confirm what was observed by NMR analysis, fraction 2 was subjected to HPLC analysis for the identification of bioactive molecules of interest. As expected, the HPLC analysis (Figure S3, Supplementary Materials) revealed the presence of sterols and carotenoids. In addition, from the HPLC data (see tables below), in this fraction, β-sitosterol (31 mg/g) and phenolic acids (111 µg/g) were about 8 and 6 times more concentrated than in crude corn oil, respectively, together with a certain amount of lutein and zeaxanthin (70 and 64 µg/g, respectively).

Despite these promising preliminary data, this type of selective extraction did not allow us to obtain good results in terms of purification of bioactive compounds. Thus, even in this case, a selective extraction of the molecules of interest was not achieved. So, we have adopted a new fractionation strategy. As reported in Section 2, three low-polarity (3, 4, 5), two medium-polarity (6, 7), and two high-polarity (8, 9) fractions were collected operating with a fractionation strategy, based on a gradient solvents system. Gravimetrical data of the obtained fractions in relation to the eluted solvents are reported in Table 1. As shown, a quantitative recovery was obtained (about 99%).

Each obtained fraction was previously analyzed by NMR spectroscopy. ^1^H and ^13^C NMR analysis of fractions 4, 5, and 6 revealed the presence of triglycerides, as also confirmed by the gas-chromatographic analysis of fractions (Table S1, Supplementary Materials). The fractions 3, 7, 8, and 9 were submitted to NMR, HPLC, and MS analyses in order to better highlight the presence and the nature of the target compounds.

**Figure 3:**
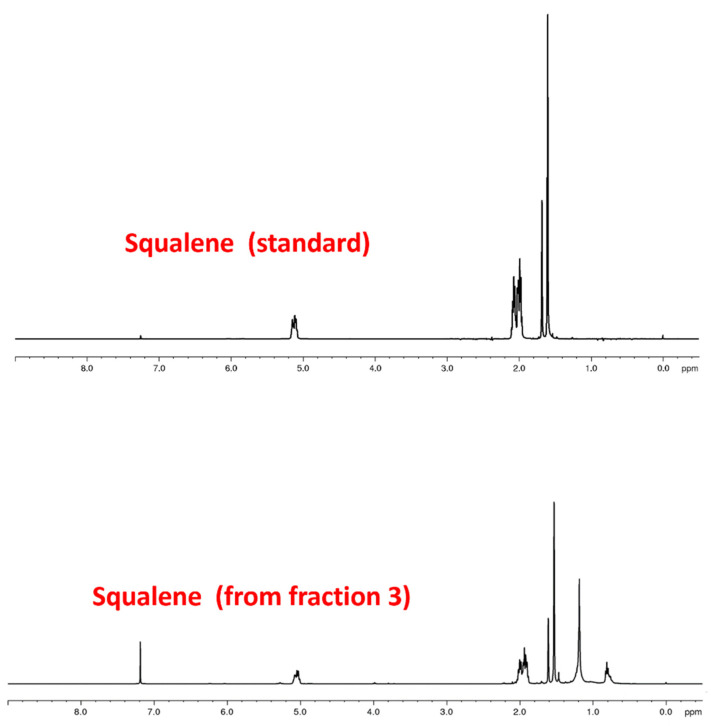
^1^H NMR spectrum of squalene obtained through fraction 3 compared with the ^1^H NMR spectrum of pure squalene.

So, although fraction 3 gave a yield of only 0.01%, the ^1^H HMR analysis revealed the prevalent presence of squalene (Figure 3), as confirmed by comparison with the spectrum of the reference standard. Considering the interest shown by this chemical specimen, the purity of the obtained extract and the exceptionally high quantities of side stream material to be managed, the squalene yield, although so low, deserves to be taken in consideration. The presence of squalene in corn oil is also confirmed in the literature [15], in which an amount of about 25 mg/100 g was observed. These data are lower compared to our results (80 mg squalene/100 g) due to the different origin of the corn oil from bioethanol production. Squalene is one of the main polyunsaturated lipids of the skin surface. It exhibits emollient and antioxidant activity and is preventive against skin cancers. It is also used as a carrier in preparations of lipid emulsions and nanostructured lipid carriers [16]. These characteristics give squalene an excellent potential in cosmetics and nutraceuticals. Based on the annual volume of side stream corn oil from ENVIRAL, the obtained putative amount of squalene could afford to about 300 Kg. The levels of sterols, tocols, and squalene were evaluated by HPLC-DAD in the obtained fractions after SPE fractionation strategy. Data are reported in Table 2, Table 3 and Table 4. Fraction 3 showed an enrichment factor of 60 with respect to crude corn oil for squalene (Table 2).

Fraction 7 had a yield of 3%, and NMR analysis had highlighted the presence of phytosterol esters along with triglycerides, so a further purification step has been performed as described in Section 2.

**Figure 4:**
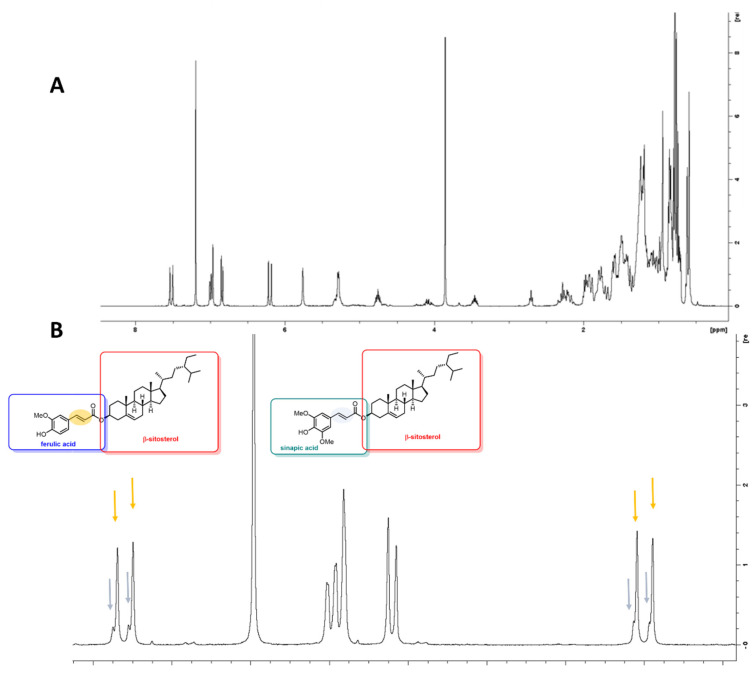
^1^H NMR of fraction 7a (Panel **A**) and ^1^H NMR of steryl-esters, olefinic region for deconvolution of fraction 7a (Panel **B**).

A white precipitate (7a) was acquired by the purification step, which analyzed by NMR and showed to be a mixture of β-sitosterol esters. More detailed evaluation on the obtained extracts revealed characteristic signals in the olefinic region of the spectrum, suggesting the presence of ferulic and sinapic esters at the position 3 of β-sitosterol. Deconvolution of the selected signals showed a ferulic/sinapic ratio of 80:20 (Figure 4).

As shown in Table 2, fraction 7 has a content of about 38 mg/g β-sitosterol with a concentration of about 10 times that of raw corn oil. Given a precipitate yield of 0.3%, one gram of corn oil side stream would yield 3 mg of an extract enriched in phytosterols as a potential food supplement. As reported in the literature, several lipid compounds such as omega-3 fatty acids and plant sterol esters have shown important efficacy in preventing cardiovascular disease [17]. Since plant sterols are also susceptible to oxidation after heat treatment, contact with oxygen, or exposure to sunlight, forming oxidation products, the presence of esters with antioxidant compounds (such as ferulic acid and synapic acid) is able to delay lipid oxidation [18]. This combination of the antioxidant and hypocholesterolemic effects of phytosterols, within this fraction, could be an interesting strategy to reduce the risk of cardiovascular disease.

Fraction 8 gave a yield of 3%. The NMR spectra of this fraction exhibit the typical signals of sterols. For this reason, a further purification step afforded a precipitate (8a) about 40 mg, whose ^1^H NMR analysis revealed the presence of β-sitosterol together with other compounds. The obtained precipitate was further purified by HPLC semipreparative and MS analysis. High purity sample of β-sitosterol, confirmed by comparison with the standard reference, was obtained as shown by Figure S4. Fraction 8 was the fraction showing the highest concentration of free sterols, mostly represented by β-sitosterol (about 60 mg/g) that here attained an enrichment factor of 14 compared to crude corn oil, but also by avenasterol, stigmasterol, campesterol, and ergosterol (enrichment factor of about 7–10) (Table 2). These results confirm ^1^H NMR analyses. No detectable amounts of carotenoids were present in this fraction. These results are in line with what reported in the literature. In fact, corn oil is considered one of the richest sources of phytosterols, especially β-sitosterol (about 300 mg/100 g of corn oil), among the most commonly used commercial oils (about 100 mg/100 g of olive oil and other vegetable oil) [19]. So, considering that these compounds reduce the absorption of cholesterol at the intestinal level, leading to a reduction in LDL plasma levels, and that their assumption could positively influence the metabolism of cholesterol [20], fraction 8 presents an excellent pharmacological potential.

**Figure 5:**
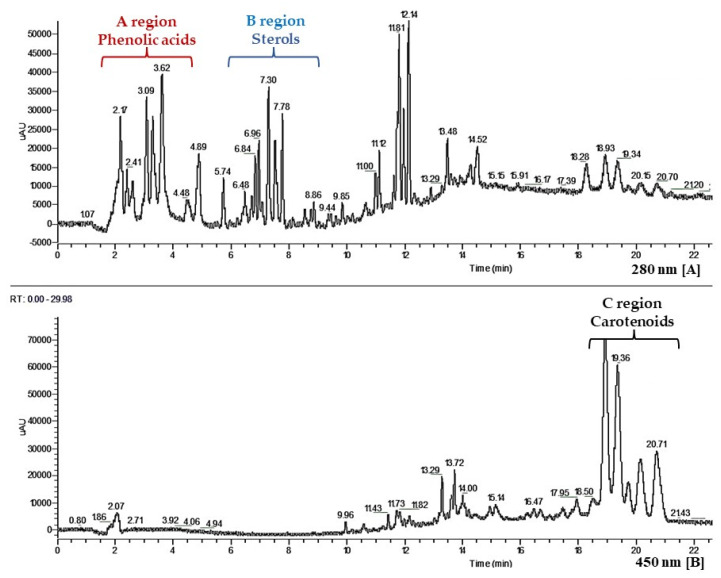
HPLC analysis of fraction 9; Panel A: Phenolics peaks (A region), Sterols peaks (B region); Panel B: carotenoids peaks (C region).

Fraction 9 (yield of 0.6%), which showed an intense reddish orange color indicating the presence of carotenoid and/or polyphenolic components, was subjected to HPLC analysis, by which three regions of interest were revealed (Figure 5).

Several classes of compounds of different polarity such as phenolic acids, among which ferulic acid, p-coumaric acid, vanillic acid, syringic acid and sinapic acid (A region, Panel A), sterols, among which ergosterol, Δ-5-avenasterol, stigmasterol, campesterol, β-sitosterol (B region, Panel A), and carotenoids, among which lutein and zeaxanthin (C region, Panel B) were identified in the mixture.

Fraction 9, the most polar fraction, showed a high concentration of sterols (enrichment factor for β-sitosterol = 10), carotenoids (enrichment factor = 5), and phenolic acids (enrichment factor = 83), confirming the indications given by qualitative HPLC analyses. The concentration of free sterols, carotenoids, and phenolics in this fraction are reported in Table 2, Table 3 and Table 4. Due to the high concentration of plant sterols (67 mg/g), phenolic acids (1.51 mg/g), as well as of carotenoids (2.39 mg/g).

For a better understanding of the molecular profile, this fraction was subjected to a further purification step. A suitable procedure has been developed and optimized for the recovery of the phytosterols and carotenoids, the most precious classes of target compounds.

Fraction 9 has been chromatographed on a 10 g RP18 silica gel, and five fractions (9a–e) have been collected as described in Section 2. These fractions were subjected to HPLC analysis, which revealed the presence of sterols and carotenoids.

The five fractions obtained after RP C18 chromatography of fraction 9 have been analyzed for their content in bioactive compounds. Results are shown in Table 2 and Table 3. Fractions 9a–e showed a high concentration of sterols in free form. Fraction 9d was the fraction with the highest concentration of β-sitosterol (enrichment factor with respect to crude corn oil of about 64), stigmasterol and campesterol (enrichment factor about 98), avenasterol (enrichment factor about 29), and ergosterol (enrichment factor about 44). Tocotrienols and tocopherols appeared not to be concentrated in these fractions (Table 2). Carotenoids were highly concentrated in fraction 9c (Table 3), where lutein and zeaxanthin in *trans* form (2573 and 2151 μg/g, respectively) together with several different *cis*-isomers attained a total carotenoid concentration of 7903 μg/g (enrichment factor about 18). These results are better than those reported in the literature (about 60–200 µg/g) [21,22] since the fractionation strategy adopted allows us to concentrate more carotenoids from the corn oil under examination.

So, fraction 9 and fraction 9c are highly rich in valuable bioactive compounds with interesting functional and antioxidant properties.

### 3.2. Antioxidant and Enzymatic Activity

In light of the results obtained, it was decided to subject the fractions richer in bioactives, 9 and 9c, to assays of antioxidant activity and enzyme inhibition.

The enriched fractions present interesting values of TPC, DPPH, TFC, and ABTS, as reported in the following Table 5. These data could be related to the presence of carotenoids compounds known for their antioxidant properties [23]. Nevertheless, it could be observed that the 9c fraction (obtained after purification of the fraction 9) has a higher antioxidant activity, confirming a higher concentration of antioxidant molecules, giving it potential as a food supplement. These results are in line with what has been reported in the literature, also with respect to other vegetable oils [24,25].

Furthermore, the enzyme inhibitory effects of the fractions (9 and 9c) were evaluated. These results are in line with the results obtained for antioxidant activities, in particular, the high activity on tyrosinase, probably due to carotenoids, gives a high potential to these enriched fractions (Table 6). These data are very important if compared with other matrices rich in carotenoids such as goji berries, which present almost comparable values (about 19 vs. 30 mgKAE/g) [23]. Additionally, anti-diabetic abilities, namely amylase and glucosidase inhibition, were higher in fraction 9c than in fraction 9. This observation could be valuable for the development of new anti-diabetic formulation by using corn oil.

## 4. Conclusions

In this work, we described the possible exploitation of corn oil from bioethanol production in high value-added applications. Our research aimed to define a valid extraction process to obtain bioactive compounds, and to this purpose, we identified solid phase extraction (SPE). Two different strategies have been investigated: 1. bind and elute strategy (BES) and 2. fractionation strategy (FS).

As the data show, the SPE-FS was the best compromise compared to the bind and elute strategy, because it resulted in fractions enriched in bioactive compounds, with particular attention to tocopherols, phytosterols, phenolic acids, and carotenoids. In addition, the proposed methodology revealed to be highly sustainable, since, through a single process, we can extract pools of different compounds with remarkable biological properties, minimizing time consumed, energy required, and solvent employed. According to a circular economy approach, the bioactive molecules in corn bioethanol co-products could be collected through appropriate technologies and re-introduced as feed, nutraceuticals, or in pharmacological or cosmetic formulations. The fractions obtained have shown a good antioxidant activity, especially the most polar fractions, rich in sterols, carotenoids, and phenolic acids, show an important activity of inhibition of tyrosinase, which makes these fractions excellent dietary supplements for the prevention and treatment of hyperpigmentation diseases. Finally, the same triglycerides, which represent more than 98% of corn oil, deserve particular attention. In fact, these could be newly employed as raw material both in the production of biodiesel and, once purified by eventual contaminants, for food, feed, or cosmetic use, minimizing the environmental impact and waste.

## Figures and Tables

**Table 1:** Gravimetric data of bioethanol corn oil fractions obtained by SPE fractionation strategy.

Fraction Polarity	Vol (L)	Mobile Phase	Yield %
3	0–0.25	*n*-hexane	0.01
4	0.25–0.50	*n*-hexane	5.90
5	0.50–1.50	*n*-hexane	44.50
6	0–1.00	*n*-hex.:Et. Acet. 85:15	41.70
7	1.00–1.50	*n*-hex.:Et. Acet. 85:15	3.10
8	0–1.00	Ethyl acetate	2.80
9	1.00–1.50	Ethyl acetate	0.60

**Table 2:** Tocols, plant sterols, and squalene in fraction 2 and in fractions 7, 8, 9, 9a–e after SPE fractionation strategy. δ-T3 was not detected in any fraction. Values are expressed as mg/g.

Fractions	γ-T3 ^a^	α-T3	δ-T	γ-T ^a^	α-T	ERG	AVN *	STG + CAMP	β-SITO	SQUA
2	-	-	-	-	-	1.54	8.79	4.71	31.39	
3	-	-	-	-	-	-	-	-	-	44.70
7	-	-	-	-	-	3.74	8.38	13.42	37.50	0.03
8	-	-	-	-	-	2.77	14.78	10.86	59.94	-
9	-	-	-	-	-	4.88	9.27	9.57	43.50	-
9a	-	-	-	-	-	-	-	-	-	-
9b	-	-	-	-	-	-	-	-	-	-
9c	-	-	-	-	-	11.28	19.24	15.70	99.37	-
9d	0.15	-	-	-	-	16.88	41.63	101.09	271.07	-
9e	0.20	0.04	-	-	-	4.17	9.95	18.79	73.56	-
Crude corn oil	0.20	0.21	0.02	0.72	0.22	0.38	1.43	1.03	4.18	0.74

^a^ may contain low or trace amounts of β-homologue. * tentative identification, quantified as brassicasterol-equivalent. Legend: T3 = tocotrienols, T = tocopherol, ERG = ergosterol, AVN = Δ − 5 avenasterol, STG = stigmasterol, CAMP = campesterol, β-SITO = β-sitosterol, SQUA = squalene. -, NOT DETECTED.

**Table 3:** Free carotenoids in fraction 2 and in high-polarity corn oil fractions 8, 9 and 9a–e after SPE fractionation strategy. Values are expressed as μg/g.

Fractions	Lutein	Zeaxanthin	N.I.C. 1	N.I.C. 2	N.I.C. 3	N.I.C. 4	N.I.C. 5	N.I.C. 6	N.I.C. 7	N.I.C. 8	N.I.C. 9	Total
2	70.1	63.6	3.9	4.4	10.9	9.7	2.7	6.5	20.5	8.6	23.5	224.4
8	-	-	-	-	-	-	-	-	-	-	-	-
9	822.0	654.0	22.8	29.3	50.5	84.2	25.9	47.1	226.2	120	304.2	2386
9a	-	-	-	-	-	-	-	-	-	-	-	-
9b	-	-	-	-	-	-	-	-	-	-	-	-
9c	2573.0	2151.0	69.1	97.5	196.8	332.5	85.1	182.3	230	1548	437.9	7903
9d	-	-	-	-	-	-	-	-	-	-	-	-
9e	-	-	-	-	-	-	-	-	-	-	-	-
Crude corn oil	133.7	134.1	3.3	7.5	10.4	26.1	13.4	-	53.6	12.4	45.4	440

N.I.C., not identified cis-isomers of carotenoids quantified as all-trans lutein-equivalent. Numbering is in order of elution time. -, NOT DETECTED.

**Table 4:** Phenolic acids detected in fraction 2 and fractions 8, 9 after SPE fractionation strategy. Values are expressed as μg/g.

Fractions	Ferulic Acid	p-Coumaric Acid	Vanillic Acid	Syringic Acid	Sinapic Acid	Sum
2	56	51	4	-	-	111
8	-	-	-	-	-	-
9	728	554	189	27	10	1508
Crude corn oil	7.83	3.89	4.54	0.91	0.89	18.06

-, NOT DETECTED (<LOD).

**Table 5:** Summary of antioxidant activity of fractions 9 and 9c.

Fractions ^1^	TPC (mgGAE/g) ^2^	TFC (mgRE/g) ^3^	DPPH (mgTE/g) ^4^	ABTS (mgTE/g) ^4^
9	14.04 ± 0.03 ^b^	3.50 ± 0.19 ^a^	5.69 ± 0.38 ^b^	14.94 ± 0.48 ^a^
9c	15.11 ± 0.10 ^a^	0.46 ± 0.07 ^b^	10.33 ± 0.27 ^a^	14.00 ± 0.05 ^b^

^1^ Values expressed are means ± S.D. of three parallel measurements. ^2^ GAE: Gallic acid equivalents; ^3^ Rutin equivalents; ^4^ TE: Trolox equivalent. Different letters (a and b) indicate significant differences in the tested fractions (*p* < 0.05).

**Table 6:** Summary of enzymatic activity.

Fractions ^1^	AChE Inhibition (mgGALAE/g) ^2^	α-Amylase Inhibition (mmolACAE/g) ^3^	α-Glucosidase Inhibition (mmolACAE/g) ^3^	Tyrosinase Inhibition (mgKAE/g) ^4^
9	0.76 ± 0.05	0.32 ± 0.02 ^b^	0.29 ± 0.02 ^a^	19.42 ± 0.96 ^a^
9c	-	0.38 ± 0.01 ^a^	0.48 ± 0.21 ^a^	16.72 ± 0.23 ^b^

^1^ Values expressed are means ± S.D. of three parallel measurements. ^2^ GALAE: Galantamine equivalent; ^3^ ACAE: Acarbose equivalent; ^4^ KAE: Kojic acid equivalent. Different letters (a and b) indicate significant differences in the tested fractions (*p* < 0.05).

## Data Availability

The data presented in this study are available in the article and in Supplementary Materials.

## Supplementary Materials

The following supporting information can be downloaded at: https://www.mdpi.com/article/10.3390/foods11020153/s1, Figure S1: ^1^H and ^13^C NMR spectra of SPE-BES fraction 1 (Panel A) and SPE-BES fraction 2 (Panel B); Figure S2: Expansion of ^13^C NMR spectra of fraction 2 related to carotenoids and sterols diagnostic region; Figure S3: HPLC analyses of fraction 2 performed at 325 and 450 nm; Figure S4: ^1^H NMR, ^13^C NMR and DI-MS of purified fraction 8a, Table S1: Fatty acid profile of low-polarity fractions (FR4-FR5) and medium-polarity fraction (FR6) obtained by SPE fractionation strategy.
