# Supplementary material for: Valorization of By-Products from Biofuel Biorefineries: Extraction and Purification of Bioactive Molecules from Post-Fermentation Corn Oil
Source: Foods. 2022 Jan 7;11(2):153. doi: 10.3390/foods11020153 (PMC8774612; doi:10.3390/foods11020153)
Supplement: Supplementary file 1 [file foods-11-00153-s001.zip › foods-1537271-supplementary.pdf]

Valorization of by-products from biofuel biorefineries: extraction and purification of bioactive molecules from post-fermentation corn oil

Francesco Cairone [1], Stefania Cesa[1], Alessia Ciogli[1], Giancarlo Fabrizi[1], Antonella Goggiamani[1], Antonia Iazzetti[1,2*], Gabriella Di Lena[3*], Jose Sanchez del Pulgar[3], Massimo Lucarini[3], Luca Cantò[4], Gokhan Zengin[5] and Petra Ondrejíčková[6]

[1] Affiliation 1; Department of Drug Chemistry and Technology, Sapienza, University of Rome, P.le A. Moro 5, 00185 Rome, Italy. francesco.cairone@uniroma1.it; stefania.cesa@uniroma1.it; alessia.ciogli@uniroma1.it; giancarlo.fabrizi@uniroma1.it; antonella.goggiamani@uniroma1.it.

[2] Affiliation 2; Dipartimento di Scienze Biotecnologiche di base, Cliniche Intensivologiche e Perioperatorie, Università Cattolica del Sacro Cuore, L.go Francesco Vito 1, 00168 Rome, Italy. antonia.iazzetti@unicatt.it.

[3] Affiliation 3; CREA Research Centre for Food and Nutrition, Via Ardeatina 546, 00178 Rome, Italy; gabriella.dilena@crea.gov.it ; jsapuri@hotmail.com ; massimo.lucarini@crea.gov.it

[4] Affiliation 4; Department of Pharmacy, University "G. d'Annunzio" of Chieti-Pescara, 66100 Chieti, Italy, luca_canto@yahoo.it.

[5] Affiliation 5; Department of Biology, Science Faculty, Selcuk University, Konya 42130, Turkey, gokhanzengin@selcuk.edu.tr

[6] Affiliation 6; ENVIRAL a.s., Trnavská Cesta, 920 41 Leopoldov, Slovakia, Ondrejickova@enviengroup.eu

* Correspondence: antonia.iazzetti@unicatt.it; gabriella.dilena@crea.gov.it

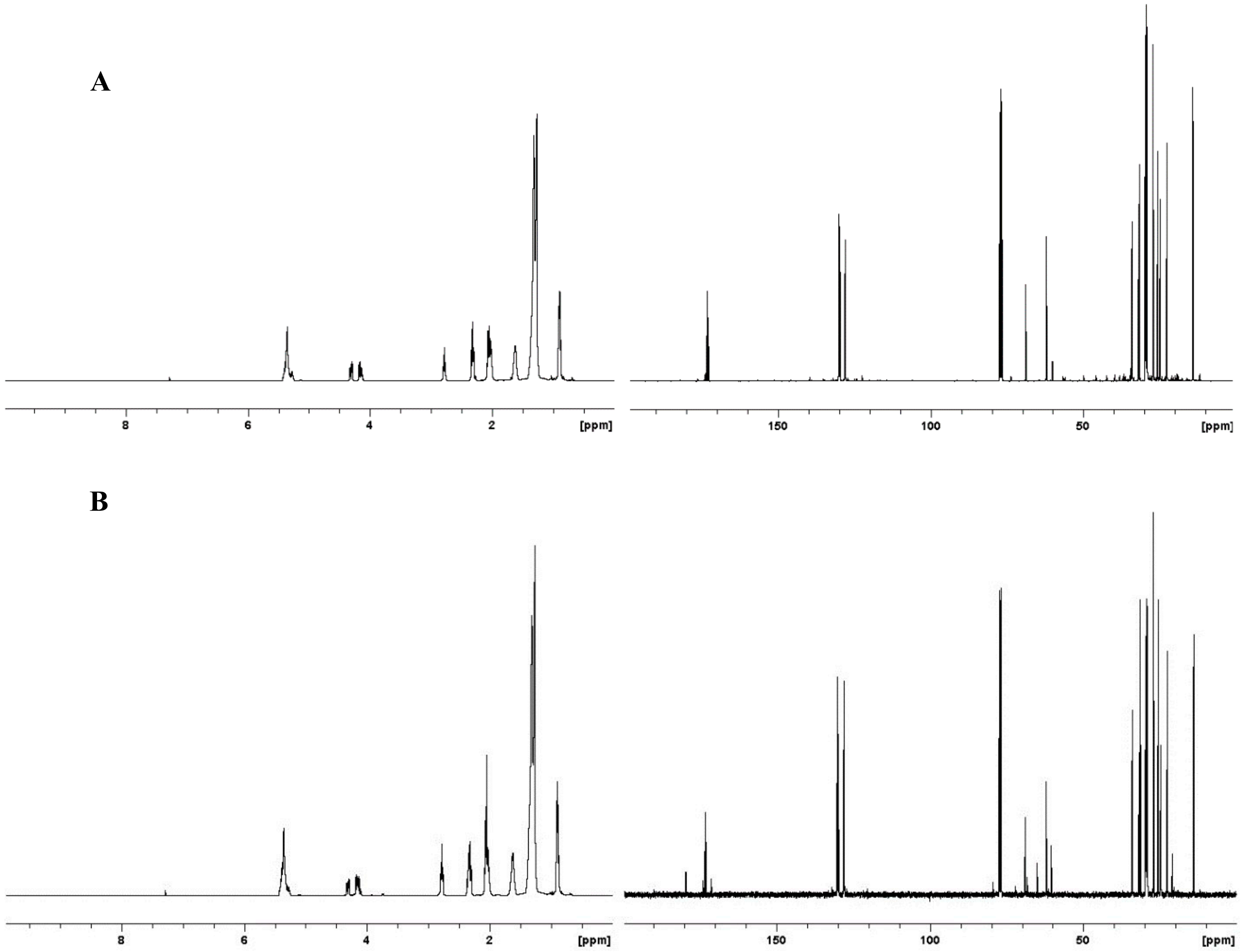

Figure S1. ^{1}H and ^{13}C NMR spectra of SPE-BES fraction **1** (Panel A) and SPE-BES fraction 2 (Panel B).

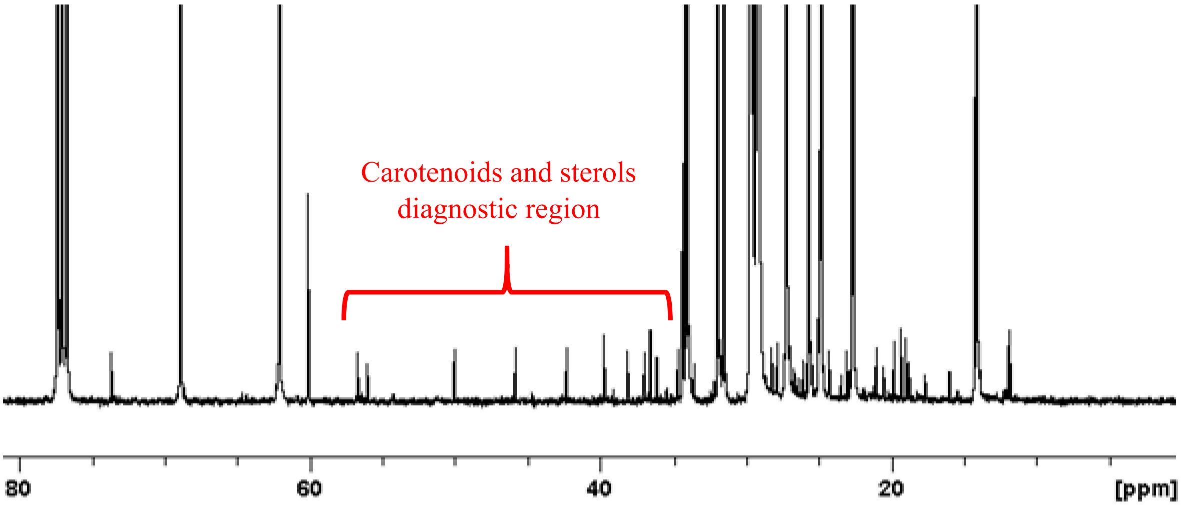

Figure S2. Expansion of ^{13}C NMR spectra of fraction **2** related to carotenoids and sterols diagnostic region.

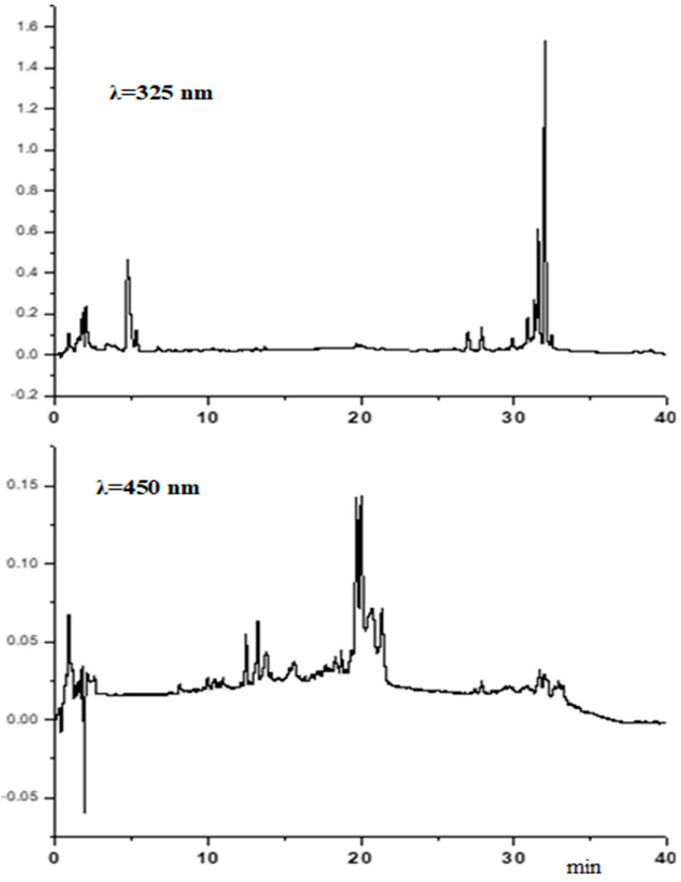

Figure S3. HPLC analyses of fraction 2 performed at 325 nm and 450 nm.

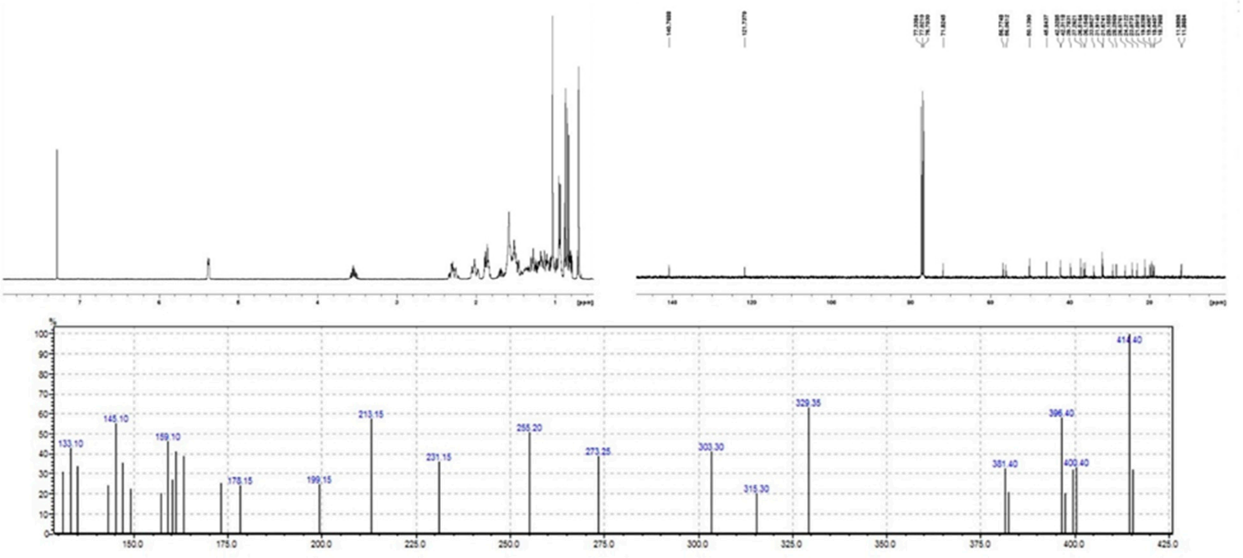

Figure S4.^{1}H NMR, ^{13}C NMR and DI-MS of purified fraction **8a.**

Table S1 Fatty acid profile of low-polarity fractions (FR4-FR5) and medium-polarity fraction (FR6) obtained by SPE fractionation strategy. Values represent % of total fatty acids.[a]

	FR 4		FR 5		FR 6	
	mean	*sd*	mean	*sd*	mean	*sd*
	% of total fatty acids					
**C12:0**	-		-		-	
**C14:0**	-		-		-	
**C15:0**	-		-		-	
**C16:0**	17.13	*0.87*	18.24	*3.53*	17.36	*3.10*
**C16:0-Ethyl ester**	3.04	*3.30*	0.78	*0.71*	-	
**C16:1 n-7**	-		-		-	
**C17:0**	-		-		-	
**C18:0**	2.43	*0.37*	3.53	*0.78*	2.43	*0.49*
**C18:1n-9**	28.22	*6.75*	27.83	*3.25*	22.28	*2.18*
**C18:1n-7**	0.75	*0.06*	0.92	*0.08*	0.73	*0.52*
**C18:1 -Ethyl ester**	1.90	*1.26*	0.58	*0.19*	-	
**C18:2 n-6**	41.18	*1.99*	44.85	*3.53*	55.54	*1.95*
**C18:2 n-6 -Ethyl ester**	4.26	*4.33*	2.32	*1.24*	-	
**C18:3 n-3**	1.06	*0.18*	0.91	*0.07*	1.78	*0.12*
**C20:0**	0.69	*0.21*	0.60	*0.04*	0.25	-
**C20:1 n-9**	0.28	*0.02*	0.33	*0.03*	0.15	-
**C20:2 n-6**	-		-		-	
**C22:0**	-		0.23	-	-	
**C24:0**	-		0.28	-	-	
**Total SFA**	23.06	*3.03*	23.32	*4.76*	19.87	*3.44*
**Total MUFA**	30.43	*5.55*	29.35	*2.81*	22.81	*1.89*
**Total PUFA**	46.50	*2.52*	47.30	*1.95*	57.31	*1.84*
**Total n-6 PUFA**	45.44	*2.38*	46.40	*1.93*	55.54	*1.95*
**Total n-3 PUFA**	1.06	*0.18*	0.91	*0.07*	1.78	*0.12*
**n-6/n-3 PUFA ratio**	43.66	*6.52*	51.27	*3.65*	31.40	*3.02*
**PUFA/SFA ratio**	2.03	*0.16*	2.10	*0.54*	2.96	*0.65*

- not detected

[a] Analyses have been carried out according to the procedure described in reference 7
